# Choice experiment selection of tourism destinations in a dual process theory framework: The role of decision style and potential to promote deliberation

**DOI:** 10.1371/journal.pone.0270531

**Published:** 2022-07-08

**Authors:** Kreg Lindberg, Kathrin Stemmer

**Affiliations:** 1 Department of Forest Ecosystems and Society, Oregon State University–Cascades, Bend, Oregon, United States of America; 2 Faculty of Environmental Sciences and Natural Resource Management, Norwegian University of Life Sciences, Ås, Norway; Fiji National University, FIJI

## Abstract

Models of consumer choice that assume rational decision processes are too simplistic, as they ignore intuitive processes and combinations of intuition and rationality. In dual process theory, System 1 processes are intuitive, fast, require low cognitive effort, and involve autonomous systems, while System 2 processes are deliberative, slower, reflect greater cognitive effort, and involve controlled attention. The dual process framework facilitates understanding of decision processes that may be diverse and complex. Based on response time as an indicator of System 2 use, we fill gaps in the tourism and choice experiment literatures by i) assessing the dimensionality of a decision style scale and its role in predicting System 2 use and ii) assessing whether researcher interventions, such as instructions, can promote System 2 use. The study is based on survey-based choice experiment responses of 483 domestic and international visitors across two Norwegian nature-based tourism destination contexts. Each visitor completed four choice experiment tasks for a total of 1,932 choice occasions. Results indicated diversity in extent of System 2 use. The decision style scale was multidimensional with both the intuitive and rational subscales predicting response time. We encourage inclusion of decision style scales–and specifically multidimensional scales–in future tourism choice and choice experiment applications. Statistically significant coefficients for instructions and unhurriedness suggest potential for researchers to increase System 2 processing in survey tasks. We encourage future use of this intervention, especially when survey tasks are intended to replicate “real world” decisions that rely heavily on System 2 use.

## 1. Introduction

There is recognition within both the applied discipline of tourism and the core discipline of economics that models of consumer choice are too simplistic when they assume deliberative and rational decision processes, ignoring intuitive processes and the combination of intuition and deliberation [1–5]. Dual process theory provides a framework for understanding diverse mental processes involved in choice. In dual process theory, System 1 processes are intuitive, fast, require low cognitive effort, and involve autonomous systems, while System 2 processes are deliberative, slower, reflect greater cognitive effort, and involve controlled attention (Evans [6] and others argue for a “type” label due to the diversity of processes that fall under System 1, but “system” is commonly used in the tourism and choice experiment fields). Dual process theory is still evolving, with competing models and recognition that decision making may involve combinations of these two archetypical process categories [6–8].

The extent to which System 2 is used for a given choice task may depend on multiple factors, including situational characteristics, such as the difficulty of the task, and personal characteristics, such as the respondent’s disposition to engage in reasoning or the respondent’s prior experience with similar choices [6, 8]. For both theoretical and practical reasons, there is value in understanding the use of System 2 processes and affecting factors.

For example, if decision style (disposition) affects System 2 use, an understanding of individual differences in decision style may facilitate customization of marketing approaches across consumers and consumption contexts. In addition, survey respondents may be less likely to engage in an extensive System 2 process in survey tasks than in the “real world” consumption decisions those survey tasks are intended to replicate. In such cases, instructions or other catalysts for System 2 use during survey tasks may enhance the accuracy of survey results relative to real world decisions.

A review of the literature indicates limited application of dual process theory in the tourism and choice experiment fields [3, 9]. Pachur and Spaar [10] used a generic vacation example in their evaluation of decision style, but we found no applications of decision response time (an indicator of System 2 use) or decision style scales in the tourism literature. The choice experiment literature includes applications of response time as a predictor of response quality [11]. One research team [12, 13] assessed decision style in the choice experiment context, but i) not within a dual process theory framework and ii) apparently using a bipolar unidimensional measure that does not allow for potential multidimensionality (varying combinations of both intuitive and rational styles).

Dual process theory is a framework for understanding choice processes. As with other theoretical frameworks, it provides a structure that can stimulate and guide research in order to build knowledge. Based on response time as an indicator of System 2 use, we fill gaps in the tourism and choice experiment literatures by i) assessing the dimensionality of a decision style scale and its role in predicting System 2 use and ii) assessing whether researcher interventions, such as instructions, can promote System 2 use. The study is based on survey choices of domestic and international visitors in two Norwegian nature-based tourism destination contexts, but the approach is relevant to other tourism and choice experiment contexts.

### 1.1. Dual process theory and choice experiments

The present study is based on the serial default-interventionist model within dual process theory [6]. In this model, the initial intuitive process (System 1) generates a default answer A1 in the face of a decision task. Deliberate processing (System 2) is then used to evaluate whether A1 is satisfactory. If A1 is evaluated as satisfactory, System 2 processing may be used to rationalize the A1 response. If A1 is evaluated as unsatisfactory, System 2 processing may be used to generate an alternative answer A2. The extent of effort during this process, as well as answer accuracy, may be affected by various factors, including respondent cognitive ability and disposition (e.g., a deliberative cognitive or decision style).

Much dual process theory research involves limited tasks with normatively correct responses and the potential for varying degrees of System 2 processing. For example, a classic task is the bat-and-ball cognitive reflection test [14]: “A bat and ball cost $1.10 in total. The bat costs $1.00 more than the ball. How much does the ball cost?” The intuitive (impulsive) answer is 10 cents, but the correct answer 5 cents. Dual process theory also has been applied in broader contexts, including choice experiments [9] and tourist consumption decisions [3].

Choice experiments are widely used in tourism and other fields to understand preferences across choice options (alternatives), to assess the importance of the attributes that characterize each option, and to estimate willingness-to-pay for attributes and packages of them [15–17]. The number of attributes and options typically is limited due to concerns about cognitive complexity, but even simple choice experiments can be more cognitively demanding than common survey tasks. An illustrative choice task involves respondents choosing across three options, with one being a neither or status quo option. The other two options are characterized by varying levels of multiple attributes.

Choice experiments differ from traditional dual process theory laboratory tasks. The consistency of responses across multiple choice experiment tasks can be evaluated [18, 19], but individual choice experiment responses are not judged normatively correct or incorrect given their dependence on respondent preferences. In addition, whereas traditional dual process theory tasks may lead to A1 responses in as few as two to three seconds [8, 20], choice experiment task response times often are longer [e.g., 11]; this leads to imperfect assessment of whether choice experiment responses reflect A1 or A2.

Task and time differences should be considered when applying a dual process theory model developed from controlled laboratory studies to choice experiment studies. Nonetheless, there is recognized diversity in tourist and choice experiment decision making processes [3, 9], with that diversity at least partly associated with extent of System 2 utilization.

The degree to which System 2 is utilized in a given decision making process is not measured directly; rather, it is assessed indirectly, if imperfectly, via indicators [8, 9, 21]. Response accuracy in cognitive reflection tests is an imperfect indicator of the extent of System 2 usage because A1 may be the normatively correct answer and A2 may be incorrect [6, 22]. Moreover, tourist trip decisions and choice experiment tasks lack the normatively correct answers commonly found in cognitive reflection tests and related tasks.

An alternative indicator is response time, the time taken to provide a response to the task. Elapsed time may be high in the System 2 evaluation and rationalization processes that lead to reporting of A1, such that longer response time potentially reflects System 2 rationalization of A1 rather than reasoning and generation of A2. Nonetheless, response time is commonly used in the psychology and, more recently, choice experiment literatures [11, 23]. Response time matches the fundamental concept of System 1 and System 2 use. As Thompson, Prowse Turner, and Pennycook [22 p 110] noted: “Given that System 2 processes are assumed to be deliberate, time consuming processes, the amount of time spent engaging in a problem should be a reliable index of the extent of System 2 processing.” Response time has been recognized as an indicator of deliberation in the choice experiment context, with response time associated with choice quality [11, 24, 25].

### 1.2. Factors potentially affecting extent of System 2 processing

Various factors potentially affect the extent of System 2 use. Though the present study is based on dual process theory, there is a related recognition of variability in response strategies in the survey research literature, using a continuum from satisficing (consistent with System 1) to optimizing (consistent with System 2).

The extent of System 2 has been linked to personal characteristics, including an individual’s inclination to engage in slow, deliberative processing [6, 26]; this is referred to as disposition or cognitive style. Several measures reflect disposition in general, including the Rational-Experiential Inventory [27] and the Need for Cognition Scale [28]. Those measures have been complemented by scales focused specifically on decision making, such as the Decision Styles Scale [29]. Within this scale, the rational (deliberative) and intuitive sub-scales have been found to be related to, but independent of, each other and to differentially correlate with outcomes across intuitive, rational, or quasi-rational task types [29; see also 30].

One might expect choice experiments to fall into the rational task category given the assumption that individuals systematically consider attributes and levels (i.e., conduct a feature-by-feature comparison [31]). However, the dependence of choices on individual preferences is consistent with intuitive tasks, and vacations are a classic example of the intuitive (experiential) category [31]. This combination of characteristics suggest tourism-oriented choice experiments may reflect a mix of both rational and intuitive styles.

De Bekker‑Grob and colleagues [12, 13] incorporated a decision style scale based on Pachur and Spaar [10] into choice experiment models in health contexts. However, their focus was not on decision style as a predictor of processing type within a dual process theory or related framework, a priority for research noted in the literature [32]. Moreover, they apparently used a unidimensional measure with intuitive and deliberative as endpoints, whereas decision style research indicates that intuitive and rational (deliberative) styles are separate dimensions, with weak to moderate correlation across the dimensions [29, 10]. Such findings argue for incorporating both dimensions as predictors.

With respect to additional personal characteristics, motivation inherent in an individual’s disposition may be complemented by motivation due to an individual’s familiarity with, and interest in, the decision task [33]. Trip choice experience may motivate deliberative processing [34]. Conversely, higher levels of experience may lead to increased perceived or actual fluency [5, 6] and thus acceptance of A1 (the initial intuitive System 1 answer) with limited additional processing.

Demographic characteristics may affect both motivation and ability. For example, IQ, education, and similar characteristics play a role in System 1, but in particular may reflect the capacity to inhibit the A1 default response and generate a new response via System 2 [8]. Cognitive ability may be particularly important in “high load” contexts, such as those involving choice experiment tasks [35]. These considerations suggest a positive correlation between education level and response time, as respondents with higher education levels may be more likely to engage in System 2 processing. On the other hand, respondents with higher education levels may complete survey tasks more quickly [36].

Likewise, there may be conflicting forces with respect to age. Individuals in higher age categories may experience decreased working memory and inhibition control, with an increased age deficit particularly impactful for complex tasks such as choice experiments [37]. This may reduce the likelihood of engaging in System 2 processing. On the other hand, the reduction in working memory capacity may lead to slower completion of survey tasks in general [36].

The selectivity hypothesis posits that females tend to process information more comprehensively than do males [38]. In the present context, this suggests longer response times for females than for males.

With respect to researcher-affected factors, there often is a goal of increasing the extent of System 2 processing (in the survey research literature: increasing optimizing over satisficing), so an important question is whether researchers can increase this extent. One potential mechanism for doing so is to inform respondents of the importance of the decision–or of engaging in deliberative processing [6, 39]. As noted in the survey literature, attention potentially can be enhanced, and satisficing behavior reduced, by “creating a sense of accountability, … asking respondents to commit to thinking carefully and generating accurate answers, and by telling respondents why the research project’s findings will be valuable and have constructive impact” [40 pp 319–320; see also 33]. This is consistent with the goal of enhancing consequentiality in stated preference studies [41]. The provision of instructions and prompts to take time and answer carefully may be effective either at the beginning of a task or interactively when speeding is detected [42].

Extensive use of System 2, either to rationalize A1 or to generate A2, typically requires more time than quick reporting of A1. Thus, the researcher goal may be to alleviate the time pressure that might limit System 2 processing [6, 8]. This is referred to here as unhurriedness.

When assessing the contribution of personal or researcher-affected factors, it is important to control for additional factors that might affect response times. Choice experiments typically involve responses to multiple choice tasks, with respondents potentially becoming more time efficient as they progress through the tasks, due to learning about the tasks and about their preferences across attributes [43]. In addition, response time (and the extent of System 2 processing) may depend on task difficulty. In the choice experiment context, the choice across options may become more difficult as the difference in utilities across the options decreases [9, 21, 43]. Thus, responses to tasks with low utility difference, which are common in optimal experimental designs, may be slower than those with high utility difference.

Though not well evaluated within a dual process framework, the screen size of the device through which the survey is completed (e.g., mobile phone, tablet, or laptop) potentially affects response time. On the one hand, smaller devices may increase the use of heuristics to simplify choice tasks that may be visually both intensive and extensive. On the other hand, the challenge of task completion on smaller devices may reduce feelings of fluency and thus catalyze increased use of slower System 2 processing [22]. In addition, task completion on smaller devices may be slower simply due to the need for additional scrolling. Research to date has not indicated a consistent relationship between device type and response quality in the context of choice tasks or surveys generally [44–46], and additional evaluation is needed with respect to the effect on response time.

Lastly, some response patterns may reflect the degree of System 2 processing and affect response time. Examples of survey satisficing behavior include straightlining or non-differentiation across multiple survey or choice tasks, with outcomes including selecting the same value across all items in a Likert scale or the same option across all choice tasks [5, 9, 40] (keeping in mind that such response patterns may reflect deliberated preferences for the presented choice tasks). Some authors consider these patterns of satisficing response patterns as reflecting reliance on heuristic cues during S1 processing, but one might alternatively view them as nonsubstantive answers rather than the substantive “going with one’s gut” type of answer consistent with System 1 as it is conceptualized in dual process theory models. Regardless, these patterns potentially reflect limited System 2 processing.

### 1.3. Research questions

The goal of this study was to extend the literature on dual process theory in tourist decision making and choice experiments, to understand diversity in choice processes, and to empirically evaluate selected aspects: the effect of personal characteristics (notably decision style), researcher-affected factors (instructions and unhurriedness), and control factors.

Evaluation of personal characteristics may help researchers and product marketers understand individual differences in tourist and choice experiment decision making. Evaluation of researcher-affected factors may help researchers increase the use of System 2 processing when conducting choice experiments. Evaluation of control factors may provide a richer understanding of System 2 processing, as well as reduce the potential effect of confounding factors.

Specific research questions are as follows, with response time serving as the indicator of System 2 processing. Expectations are based on previous research, described in Section 1.2.

*1a. Is respondent decision style (disposition) in the tourism choice context multidimensional?*

Decision style is expected to be multidimensional, with separate factors (dimensions) for the intuitive and rational subscales.

*1b. Do both intuitive and rational dimensions of decision style predict System 2 processing?*

Intuitive decision style is expected to negatively correlate with response time, while rational decision style is expected to positively correlate with response time.

*2. Do instructions and respondent unhurriedness predict System 2 processing?*

Instructions and unhurriedness are expected to increase response time.

*3. Do past trip experience, education, age, and gender (personal characteristics beyond decision style) predict System 2 processing?*

Female respondents are expected to have longer response times. Due to potential countervailing effects, there are no *a priori* expectations about the other factors.

*4. Do task order, utility difference, screen size, and satisficing behavior (selecting the same option across all tasks) predict System 2 processing?*

Later tasks are expected to involve less response time than earlier tasks. Tasks with larger utility difference are expected to involve less response time than those with smaller utility difference. Due to potential countervailing effects, there is no *a priori* expectation about the effect of screen size. Satisficing behavior is expected to reduce response time.

## 2. Materials and methods

### 2.1. Context

**Fig 1:**
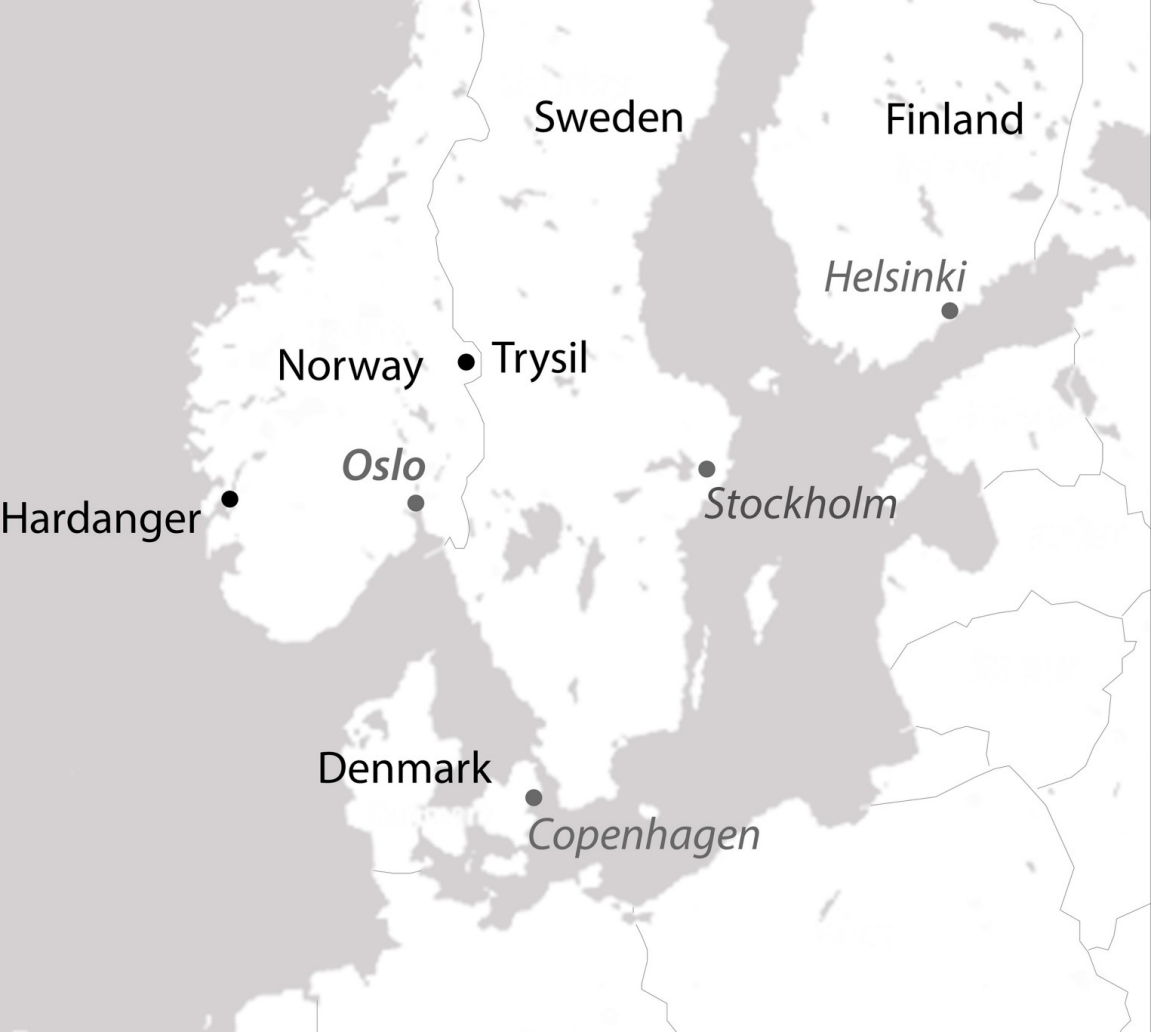
Study area.

This study was based on surveys of nature-based tourism visitors to the Trysil and Hardanger areas of Norway (Fig 1). Trysil is primarily a winter destination in the eastern part of Norway, with one of the larger downhill ski areas in Scandinavia. The region is expanding summer season visitation through development of fishing and mountain biking opportunities; mountain biking was the focus of the choice experiment among Trysil visitors. Hardanger is primarily a summer destination in western Norway that is widely known for its mountains, glaciers, waterfalls, fjords, and fruit farms. Trolltunga (the troll’s tongue) has become an iconic hiking destination due to tourist-generated photos on social media; it attracted 90,000 visitors in 2019 [47]. Hiking was the focus of the choice experiment among Hardanger visitors.

### 2.2. Methods and measures

The surveys included choice experiments of destination package choice, with activities representing a primary attraction at each site. The Norwegian Centre for Research Data (NSD) evaluated the handling of personal data in this project (project numbers 54755, 54756 & 58311) as being in accordance with data protection legislation.

Study participants were recruited on-site with a short self-administered registration survey during the summer season of 2017. They were then invited to complete the full survey online in 2018. Response rates for these on-site samples were 52 percent for Trysil (mountain bikers) and 45 percent for Hardanger (hikers).

In addition, the tourism board in Trysil (Destinasjon Trysil) asked persons on their mailing list (N = 10,591) to complete the online survey if they mountain biked in Trysil in 2017. A total of 847 (8 percent) responded, but only 383 of these were confirmed as bikers. Of those, 224 (58 percent) completed the survey. A drawing-based incentive of 1000 euros was provided for survey completion, applied to the on-site and Destinasjon Trysil samples combined.

For the choice experiments, survey participants were asked to assume they were planning their next trip and deciding whether to book a package for the trip. We then presented participants with introductory information and four sequential choice tasks comprised of two alternative packages, each containing specified levels of characteristics (attributes). We asked participants to assume they only had a choice between the two presented package options or a neither (opt out) option. The choice experiment results are reported elsewhere [48], but an illustrative choice task (Table 1) and the set of attributes and levels (Table 2) are presented here to illustrate the tasks.

**Table 1:** Illustrative choice experiment task.

Attribute	Biking Package 1	Biking Package 2
Trail diversity	Multi-user trails (not specifically designed for MTB)	Multi-user and purpose-built MTB trails
Biking skills course OR guided biking tour (half-day)	Not included / biking on your own	Small group biking tour
Additional guided tour (optional, non-biking, half-day)	Choice of nature tour or cultural tour included	Not included
Accommodation	Budget 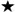	Comfort 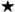 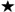 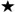
Eco-certification of package	All service providers are eco-certified	Service providers are NOT eco-certified
Package price per night (covers course, guided tours, life and accommodation)	75€ for 1 person, 100 for 2 persons	300€ for 1 person, 375 for 2

**Table 2:** Choice experiment attributes and levels.

Attribute	Mountain biking (Trysil)	Hiking (Hardanger)
Quality	Some biking sites offer only multi-user trails, while others also offer purpose-built MTB trails, cross-country and downhill trails, and pump tracks/jump lines. 1. Multi-user trails (not specifically designed for MTB). 2. Multi-user and purpose-built MTB trails.	The length of the hike in hours, excluding breaks. Hiking is on trails/paths in mountainous terrain with limited signage or trail marking. 1. 1–4 hours. 2. 4–8 hours. 3. 8–12 hours.
Guiding	Biking skills course or half-day guided biking tour. The course and tour are customized to individual skill level, organized in small groups (maximum 8 persons) and led by professional instructors/guides. 1. Not included. Biking on your own. 2. Small group biking skills course. 3. Small group biking tour.	Professional guided tour. Small groups (maximum 8 persons) led by qualified guides that know the area well and have first aid, wilderness, navigation and interpretation skills. 1. Not included. Hiking on your own. 2. Professional guide services included.
Tours	Additional half-day guided tour available. Nature-oriented tours involve visiting natural attractions and/or doing activities in nature. Cultural-oriented tours involve local history, culture and/or local foods. 1. Not included. 2. Choice of nature tour or cultural tour included.	
Lodging	Accommodation level. Budget offers very basic services and amenities with a shared bathroom. Comfort offers good quality hotel services and amenities with private bathroom. Luxury offers premium quality services and amenities. 1. Budget 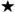 2. Comfort 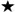 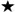 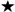 3. Luxury 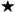 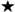 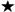 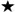 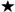	
Eco-certification	Some packages make use of eco-certified service providers, guaranteeing minimal emissions and impact on the surrounding environment, with maximum use of local food/services. Others do not use eco-certified providers. 1. Service providers are NOT eco-certified. 2. All service providers are eco-certified.	
Price	Package price per night. Includes lodging, breakfast, course, guided tours and lift fees, where applicable. 1. 75€ for 1 person, 100 for 2 2. 150€ for 1 person, 200 for 2 3. 300€ for 1 person, 375 for 2 4. 500€ for 1 person, 600 for 2	Package price per night. Includes lodging, breakfast and guide services, where applicable. 1. 75€ for 1 person, 100 for 2 2. 150€ for 1 person, 200 for 2 3. 300€ for 1 person, 375 for 2 4. 500€ for 1 person, 600 for 2

Attribute description and levels with merged columns were the same across the two sites.

A d-efficient experimental design with 24 choice sets was created in Ngene version 1.2 [49] and used to allocate attribute levels across choice options in those sets. The 24 sets were blocked into six questionnaire versions that were randomly allocated across respondents; each respondent selected one option for each of four presented choice sets.

The primary analysis was of response time as a function of personal characteristics, researcher-affected characteristics, and controls. The variables are described here, with survey wording presented in Tables 3 and 4.

**Table 3:** Variables and descriptive statistics.

Variable description	Mean	SD
*Response time*, in seconds, natural log.	3.05	0.85
*Intuitive decision style (disposition)*, mean of the three items shaded in the second factor in Table 4, 1 = strongly disagree to 7 = strongly agree, with the following introductory wording: Consider the trips you make that involve an overnight stay away from home to engage in biking / hiking or other outdoor activities. To what extent do you agree or disagree with the following statements in the context of your decisions about where to go and what to do for such trips?	4.49	1.12
*Rational decision style (disposition)*, mean of the three items shaded in the first factor in Table 4, presented together with items for intuitive decision style.	5.05	1.09
*Trip experience*, mean of the following two items, divided by 10.	0.57	0.41
At how many different sites did you go biking / hiking last year (2017)?		
How many years in total have you been biking / hiking?		
*Education*, dummy variables for 1 to 4 years of university (40% of sample) and for graduate degree / more than 4 years of university (34%).		
*Gender*, dummy variable indicating female (45% of sample).		
*Age*, dummy variables for respondents in their 30s (19% of sample), 40s (42%), 50s (15%), and 60 or older (3%).		
*Instructions*, dummy variable (50% of sample) for presence of following wording: Please take your time answering these questions, as biking / hiking areas like Trysil / Trolltunga (Hardanger) may make investment and management decisions based on survey responses.		
*Unhurriedness*, response on scale of 1 = Hurried to 7 = Unhurried (relaxed), with the following introductory wording: We would like to understand how you felt during the time you completed this survey–with your feelings possibly being affected by the survey itself or by other things happening in your life. Would you say you felt…?	5.49	1.53
*Order*, dummy variables for second, third, and fourth choice task (each 25% of the sample).		
*Utility difference*, based on attributes-only choice model for each site.	0.52	0.36
*Screen size*, resolution of longest side, divided by 100.	11.64	5.32
*All option 1 or 2*, dummy variable (11% of sample).		
*All neither option*, dummy variable (5% of sample).		

**Table 4:** Decision style scale factor analysis.

Item wording	Loadings	
	Rational	Intuitive
I gather all the necessary information before I make these decisions.	0.83	-0.05
When I make these decisions, I mainly rely on my gut feelings.	0.00	0.82
I weigh feelings more than analysis in making these decisions.	-0.08	0.79
I thoroughly evaluate alternative locations before making final choices.	0.84	-0.07
I rely on my first impressions when making these decisions.	0.03	0.80
I weigh several different factors when making these decisions.	0.82	0.07
Cronbach’s alpha, shaded items	0.77	0.73

Method = principal components and varimax rotation.

The respondent-level variables (intuitive decision style, rational decision style, trip experience, education, gender, age, instructions, unhurriedness, screen size, all option 1 or 2, and all neither option) reflect between-subjects data. The choice-level variables (response time, order, and utility difference) reflect within-subjects data. The decision style analysis (results in Table 4) reflects between-subjects data, with one observation per respondent. The model of factors predicting response time (results in Table 5) reflects a combination of between- and within-subjects data, with four observations per respondent (one observation for each of the four choices made by each respondent).

**Table 5:** Model of factors predicting response time.

Variable	Coefficient (95% CI)	SE	P-value
Intercept	3.468 (2.997–3.939)	0.240	< 0.001
Intuitive style	-0.086 (-0.130 –-0.042)	0.022	< 0.001
Rational style	0.055 (0.008–0.102)	0.024	0.022
Trip experience	0.072 (-0.052–0.195)	0.063	0.258
Education—university	-0.057 (-0.192–0.078)	0.069	0.410
Education—graduate degree	-0.078 (-0.219–0.062)	0.072	0.275
Gender–female	0.156 (0.050–0.261)	0.054	0.004
Age— 30s	0.061 (-0.077–0.199)	0.070	0.386
Age— 40s	0.032 (-0.093–0.156)	0.063	0.618
Age— 50s	0.097 (-0.070–0.264)	0.085	0.256
Age—60+	0.200 (-0.075–0.474)	0.140	0.153
Instructions	0.131 (0.029–0.233)	0.052	0.012
Unhurriedness	0.038 (0.000–0.075)	0.019	0.050
Order— 2^nd^	-0.490 (-0.554 –-0.427)	0.032	< 0.001
Order— 3^rd^	-0.792 (-0.860 –-0.724)	0.035	< 0.001
Order— 4^th^	-0.967 (-1.037 –-0.898)	0.035	< 0.001
Utility difference	-0.080 (-0.152 –-0.008)	0.037	0.029
Screen size	-0.013 (-0.022 –-0.004)	0.005	0.006
All option 1 or 2	-0.388 (-0.578 –-0.198)	0.097	< 0.001
All neither option	-0.531 (-0.930 –-0.133)	0.203	0.009

The choice experiment introduction and each of the four choice tasks were presented on separate screens. Response time is the elapsed time in seconds from i) when the respondent clicked to proceed to the screen to ii) when the respondent clicked to proceed to the next screen. Thus, the response time for the second task (Order 2^nd^) was the elapsed time between clicking the continue arrow at the bottom of the first choice task screen to clicking the continue arrow at the bottom of the second choice task screen.

Because decision style (disposition) may vary across decision objects [10], intuitive and rational decision styles were assessed in the specific context of previous overnight trips to engage in biking (for Trysil) or hiking (for Hardanger), using a modified Decision Styles Scale [29]. Item wording is provided in Table 4, and items were presented in random order across respondents. Respondents could opt out of this question if they had not made such trips or had not participated in decision making for trips they had made. Such respondents were not included in this analysis.

Trip experience was measured as the mean of i) the number of sites at which the respondent had participated in biking (for Trysil) or hiking (for Hardanger) in the previous year and ii) the number of years the respondent had engaged in biking or hiking. Education, gender, and age were analyzed as dummy variables, with males and the lowest level of education and age used as reference categories.

The choice experiment task included an introduction, with half the respondents selected at random being presented an additional paragraph specifically requesting that respondents take their time because the destinations may make investment and management decisions based on their responses. In addition, we asked respondents to report their affect while completing the survey, using a semantic differential format. One of the five word pairs was hurried and unhurried (relaxed).

Several variables were used as controls. The order within the set of four choice tasks was recorded for each task. Utility difference calculation followed Olsen et al. [50] and was based on attributes-only models and the difference in calculated utilities between the two options with highest utility; this included the opt out option if one or both “action” options had negative utility. Screen size was the resolution of the longest side of the device on which the survey was completed, as indicated by the online survey paradata. Dummy variables were created to indicate whether respondents selected i) either all option 1 or all option 2 or ii) all neither option.

### 2.3. Data and analyses

The trip experience and screen size variables were transformed (divided by 10 and 100, respectively) to preserve two significant digits in the presentation of coefficients (Table 5). The distribution of choice task response times exhibited an extended right tail, with 13% of observations being 600 seconds (10 minutes) or more. Following Lohse, Goeschl, and Diederich [25], we assumed that very high-duration observations reflected respondents leaving the task temporarily rather than engaging in particularly extensive System 2 processing; they were thus treated as invalid observations and removed. After removal of observations greater than or equal to 600 seconds, the response time distribution remained skewed. Therefore, the natural log of response time was used for the response time variable.

A conservative listwise approach was utilized, with all four choice observations for a respondent deleted if there was a missing value for any variable in the model. For example, all four observations were removed if a respondent did not report education or spent more than 600 seconds on one of the four choice tasks. This led to 1,232 choice observations (308 respondents) for Trysil and 700 choice observations (175 respondents) for Hardanger.

The response time model was analyzed with the Mplus version 8 [51] two-level routine to account for the panel nature of the data (the combination of between- and within-subjects data). All other analyses were conducted using SPSS version 26.

## 3. Results

Exploratory factor analysis results for the modified Decision Styles Scale are shown in Table 4. There was simple structure, with two distinct factors and Cronbach’s alpha values similar to those in [29] despite the reduced number of items per factor. The loading pattern indicates that respondent decision styles were multidimensional, rather than falling along a single bipolar continuum from intuitive to rational. The Pearson correlation between the two decision style measures was -.038 (N = 483 respondents, p = 0.402), also consistent with multidimensionality.

Model results are presented in Table 5, with the natural log of response time in seconds as the dependent variable, unstandardized coefficients, two-tailed p-values, and 95% confidence intervals. Trip experience, education, and age were nonsignificant at α = 0.05. All other variables were significant predictors of response time. The R^2^ for the choice level (utility difference and the three order variables) was 0.33. The R^2^ for the respondent level (all other variables) was 0.19. Thus, a combination personal characteristics, researcher-affected factors, and task characteristics is relevant for understanding, and explaining variation in, response time.

Response time increased with increasing levels of rational decision style and decreasing levels of intuitive decision style. Females tended to spend more time responding. Time on task increased with motivational instructions and when respondents felt unhurried.

With respect to task characteristics, respondents spent less time on each choice as they proceeded through the four choice tasks. They also spent less time on choice tasks involving larger utility differences. Respondents completing the choice tasks with a larger device did so more quickly than those with smaller devices. Lastly, respondents selecting all the same option completed the tasks relatively quickly.

## 4. Discussion and conclusions

This study responds to the call for empirical evaluation of the dual process framework in the context of tourism and broader choice contexts [3, 5], as well as for assessing the role of decision style in dual process and choice experiment contexts [13, 32]. We applied dual process theory, using response time as an indicator of extent of System 2 processing, to understand diversity in choice processes and the contributions to that diversity of decision style (disposition) and other personal characteristics, researcher-affected factors, and control factors.

With respect to the four research questions (Section 1.3), results were consistent with all prior expectations for coefficient signs, which were based on previous research. For Research Question 1a, a modified version of the Decision Styles Scale [29] had good psychometric properties and a multidimensional structure, with separate factors for the intuitive and rational subscales. Whereas previous research [29, 10] found weak to modest correlation between subscales, the present study found weak and nonsignificant correlation (Pearson coefficient = -.038, p = .402). This multidimensionality is consistent with the dual process conception of respondents using various combinations of both System 1 and System 2.

For Research Question 1b, both decision style subscales significantly predicted response time, with respondents high in intuitive decision style completing choice tasks relatively quickly and respondents high in rational decision style taking more time. This was consistent with the expectation that respondents high in rational decision style engage in more System 2 processing than do those low in rational decision style. The present study appears to be the first to use a decision style scale in a specific tourist choice context (a generic vacation served as one of the six domains evaluated in Pachur and Spaar [10]) and the first to use a multidimensional decision style scale in the choice experiment field.

With respect to Research Question 2, unhurriedness and instructions to increase motivation and System 2 processing both predicted response time. While recognizing the limits of encouraging System 2 use in non-laboratory tasks, results were consistent with the broader literature indicating the potential to increase deliberative processing through instructions [6, 40]. Moreover, online survey functionality facilitates researcher interventions beyond simply providing instructions, such as via queries or prompts when speeding or inconsistent response patterns are detected [42].

The present study did not manipulate unhurriedness when completing the survey, but results suggest the potential benefit of researchers encouraging respondents to complete the survey when they have time to do so in a relaxed manner. Researchers may facilitate this goal by ensuring the duration of the overall survey, or of the choice task component specifically, is reasonable given the context (the motivation of potential respondents, the nature of any incentives, and so on).

With respect to the role of personal characteristics beyond decision style (Research Question 3), female respondents utilized more time in completing the choice tasks, consistent with the selectivity hypothesis [38]. Coefficients for other personal characteristics were nonsignificant, possibly due to the countervailing factors noted in the introduction. With respect to control factors (Research Question 4), various task characteristics predicted System 2 processing. As expected, respondents completed choice tasks later in the sequence more quickly than those earlier in the sequence [43]. Likewise, larger utility differences were associated with quicker response times, consistent with larger differences leading to the appeal of one option being more quickly apparent [9, 21, 43].

### 4.1. Contributions and implications

This study fills a gap in the literature and introduces a multidimensional decision style scale to the tourism and choice experiment fields. Present results indicate the importance of using multidimensional measures in future studies.

In addition, the study illustrated how researchers may be able to promote System 2 use in survey contexts. We do not assume that System 2 is inherently better than System 1, but we expect that some “real world” decisions are more likely than others to involve System 2 processing. Insofar as research is intended to replicate “real world” decisions, such as to generate willingness-to-pay estimates for policy making, interventions such as instructions potentially increase System 2 use in the survey context to better match System 2 use in “real world” contexts–thereby contributing to more informed policy making.

Most fundamentally, this study contributes to the tourism and choice experiment literatures by extending the promising, but so far relatively limited, use of dual process theory. Personal characteristics have been a significant focus of the tourist decision making and marketing literature [3, 52], and those characteristics remain relevant when applying dual process theories. However, the dual process approach provides a framework for understanding the mechanisms through which personal characteristics (as well as researcher interventions and control factors) may affect cognitive processes and associated choices.

The specific inclusion of a decision style scale when evaluating tourist decisions can enhance both theory development and the effectiveness of destination marketing by helping illuminate the “black box” of the diverse processes involved in decision making, thereby allowing more inclusive and customized tourism marketing approaches. For example, the mix of marketing approaches can be customized to potential customers who make decisions quickly and with presumed reliance on System 1 processes, those who engage in substantial information processing (System 2), and those with varying combinations of these approaches. As McCabe, Li, and Chen [3 p 12] note, marketing messages across different contexts can be aligned with decision processes; they observe that avenues for future research include the “timing of campaigns, type of information provided, pricing strategy, and use of discounts and promotions to elicit specific types of decision strategies.”

### 4.2. Limitations and future research

This study focused on dual process theory in the specific context of a survey-based choice experiment of nature-based tourism destination choice in Norway. We encourage replication to better understand how System 2 use, and the factors predicting it, varies across choice contexts, including across diverse types of tourist choices and across non-tourist choices. Relative to the present trip choice context, some tourist decisions may rely more heavily on System 1 processing, with the intuitive decision style playing a positive role. Conversely, other decisions may rely more heavily on System 2, with the rational decision style playing a positive role. It will be important for future research to assess and model decision style in a multidimensional format unless a unidimensional format is indicated, such as by a single-factor solution in exploratory factor analysis.

The inclusion of decision style as a predictor of System 2 use fills a gap in the literature. However, substantial variance in response time remains to be explained. Therefore, we encourage further exploration of potential predictors to explain individual differences in System 2 use.

The present study focused on response time as the outcome, rather than as a predictor of response quality in choice experiments. In the latter context, response time has been used in various forms, including as a criterion for membership in latent classes [11], while predictors of response time have been evaluated separately in some studies [e.g., 24]. Hybrid choice models provide the opportunity to incorporate response time as both an outcome (predicted by personal and task characteristics) and a predictor of response quality [53, 54]. To date, such analyses have focused on demographic characteristics as predictors of response time, and we encourage inclusion of decision style.

Choice experiments are often conducted via surveys, with interest in response time motivated in part by concern that estimates of preferences and willingness-to-pay may be affected by “professional” respondents in internet panels [54]. There is substantial laboratory-based evaluation of dual process theory in psychology [55], and we encourage similar non-survey analyses within choice experiment and tourism contexts.

## Supporting information

S1 Data (XLSX)
